# Progress Toward Rubella Elimination — World Health Organization European Region, 2005–2019

**DOI:** 10.15585/mmwr.mm7023a1

**Published:** 2021-06-11

**Authors:** Patrick O’Connor, Dragon Yankovic, Laura Zimmerman, Myriam Ben Mamou, Susan Reef

**Affiliations:** ^1^Vaccine Preventable Disease and Immunization Flagship Programme, World Health Organization European Regional Office, Copenhagen, Denmark; ^2^Global Immunization Division, Center for Global Health, CDC.

In 2005, the Regional Committee of the World Health Organization (WHO) European Region (EUR) passed a resolution calling for the regional elimination of measles, rubella, and congenital rubella syndrome (CRS) (*1*). In 2010, all 53 countries in EUR* reaffirmed their commitment to eliminating measles, rubella, and CRS (*2*); this goal was included in the European Vaccine Action Plan 2015–2020 (*3*,*4*). Rubella, which typically manifests as a mild febrile rash illness, is the leading vaccine-preventable cause of birth defects. Rubella infection during pregnancy can result in miscarriage, fetal death, or a constellation of malformations known as CRS, which usually includes one or more visual, auditory, or cardiac defects (*5*). The WHO-recommended measles and rubella elimination strategies in EUR include 1) achieving and maintaining ≥95% coverage with 2 doses of measles- and rubella-containing vaccine (MRCV) through routine immunization services; 2) providing measles and rubella vaccination opportunities, including supplementary immunization activities (SIAs), to populations susceptible to measles or rubella; 3) strengthening surveillance by conducting case investigations and confirming suspected cases and outbreaks with laboratory results; and 4) improving the availability and use of evidence to clearly communicate the benefits and risks of preventing these diseases through vaccination to health professionals and the public (*6*). This report updates a previous report and describes progress toward rubella and CRS elimination in EUR during 2005–2019 (*7*). In 2000, estimated coverage with the first dose of a rubella-containing vaccine (RCV1) in EUR was 60%, and 621,039 rubella cases were reported (incidence = 716.9 per 1 million population). During 2005–2019, estimated regional coverage with RCV1 was 93%–95%, and in 2019, 31 (58%) countries achieved ≥95% coverage with the RCV1. During 2005–2019, approximately 38 million persons received an RCV during SIAs in 20 (37%) countries. Rubella incidence declined by >99%, from 234.9 cases per 1 million population (206,359 cases) in 2005 to 0.67 cases per 1 million population (620 cases) by 2019. CRS cases declined by 50%, from 16 cases in 2005 to eight cases in 2019. For rubella and CRS elimination in EUR to be achieved and maintained, measures are needed to strengthen immunization programs by ensuring high coverage with an RCV in every district of each country, offering supplementary rubella vaccination to susceptible adults, maintaining high-quality surveillance for rapid case detection and confirmation, and ensuring effective outbreak preparedness and response.

## Immunization Activities

Since 2009, all 53 countries in EUR have included 2 RCV doses as part of a combination vaccine with measles and mumps (MMR)^†^ or measles, mumps, and varicella (MMRV) vaccine in their routine childhood vaccination schedules. WHO and the United Nations Children’s Fund (UNICEF) estimate annual vaccination coverage for all countries in the region using government-reported administrative coverage data (calculated as the number of doses administered divided by the estimated target population) and vaccination coverage surveys. During 2005–2019, estimated regional coverage with RCV1 was 93%–95%, and in 2019, 31 (58%) countries achieved ≥95% coverage with the first dose of RCV. In 2005, estimated regional coverage with the second dose of RCV was 76%; and in 2019, second dose coverage had risen to 91% (Table). In 2019, estimated national RCV2 coverage ranged from 76% to 99%. During 2005–2019, >38 million persons received RCV in 71 SIAs conducted in 20 (37%) countries.

**TABLE:** Year of introduction, age at vaccination, and estimated coverage with the first and second doses of rubella–containing vaccine,* and number of rubella cases^†^ and incidence,^§^ and congenital rubella syndrome cases, by country — World Health Organization European Region, 2005, 2015, and 2019

Country (year RCV introduced into routine immunization schedule)	2019 RCV schedule, age^¶^		2005				2015				2019			
			% Coverage		No. of rubella cases (incidence)^§^	No. of CRS cases	% Coverage		No. of rubella cases (incidence)^§^	No. of CRS cases	% Coverage		No. of rubella cases (incidence)^§^	No. of CRS cases
	1st dose	2nd dose	RCV1	RCV2			RCV1	RCV2			RCV1	RCV2		
Albania (2001)	12 mos	5 yrs	97	97	0 (—)	0	97	98	0 (—)	NR	95	**96**	0 (—)	0
Andorra (1988)	12 mos	3 yrs	94	NR	0 (—)	0	96	88	0 (—)	0	99	95	0 (—)	0
Armenia (2002)	12 mos	6 yrs	94	92	620 (207.9)	NR	97	97	0 (—)	0	95	96	0 (—)	0
Austria (1973)	9 mos	+1 mo after 1st dose	75	54	NR	NR	96	88	0 (—)	0	94	84	0 (—)	NR
Azerbaijan (2003)	12 mos	6 yrs	67	67	1,025 (120.0)	0	98	98	0 (—)	0	98	97	2 (0.2)	1
Belarus (1996)	12 mos	6 yrs	99	98	3,812 (398.6)	NR	99	99	1 (0.1)	0	98	98	0 (—)	0
Belgium (1985)	12 mos	10–12 yrs	88	NR	NR	0	96	85	0 (—)	0	96	95	0 (—)	0
Bosnia and Herzegovina (1980)	12 mos	6 yrs	90	90	43 (11.4)	0	83	88	12 (3.1)	NR	68	76	3 (0.9)	NR
Bulgaria (1993)	13 mos	12 yrs	96	92	1,968 (256.0)	0	92	87	0 (—)	5	93	87	0 (—)	0
Croatia (1975)	12 mos	4–6 yrs	96	98	3 (0.7)	0	93	96	0 (—)	0	93	95	0 (—)	0
Cyprus (1974)	12–15 mos	4–6 yrs	86	NR	0	0	90	88	2 (1.7)	0	86	88	0 (—)	0
Czechia (1983)	13 mos	5 yrs	97	98	8 (0.8)	0	99	99	1 (0.1)	0	92	94	0 (—)	0
Denmark (1987)	15 mos	4 yrs	95	91	0 (—)	0	91	80	0 (—)	0	96	90	0 (—)	0
Estonia (1992)	12 mos	13 yrs	96	98	6 (4.4)	0	93	92	0 (—)	0	88	90	0 (—)	0
Finland (1975)	12 mos	6 yrs	97	NR	0 (—)	0	95	93	10 (1.5)	1	96	93	0 (—)	0
France (1983)	12 mos	18 mos	87	NR	NR	NR	91	79	0 (—)	2	90	83	0 (—)	NR
Georgia (2004)	12 mos	5 yrs	90	87	1,841 (437.3)	1	96	91	100 (25.0)	0	99	97	9 (2.3)	0
Germany (1980)	11–14 mos	15–23 mos	96	91	NR	0	97	93	91 (1.1)	0	97	93	56 (0.7)	0
Greece (1995)	12–15 mos	2–3 yrs	96	NR	16 (1.4)	0	97	83	0 (—)	0	97	83	0 (—)	0
Hungary (1989)	15 mos	11 yrs	99	99	32 (3.2)	0	99	99	0 (—)	0	99	99	0 (—)	0
Iceland (1979)	18 mos	12 yrs	90	90	0 (—)	0	93	94	0 (—)	0	93	95	0 (—)	0
Ireland (1971)	12 mos	4–5 yrs	84	NR	17 (4.1)	0	93	91	9 (1.9)	0	92	90	0 (—)	0
Israel (1995)	12 mos	6 yrs	94	96	23 (3.5)	0	98	97	1 (0.1)	0	98	96	0 (—)	NR
Italy (1990)	13–15 mos	6 yrs	87	NR	171 (2.9)	NR	85	83	39 (0.6)	0	94	88	22 (0.4)	0
Kazakhstan (2004)	12 mos	6 yrs	99	96	8,783 (570.2)	0	99	98	2 (0.1)	0	99	98	5 (0.3)	0
Kyrgyzstan (2001)	12 mos	6 yrs	99	98	1 (0.2)	0	99	96	100 (16.8)	0	96	98	2 (0.3)	0
Latvia (1993)	12 mos	7 yrs	98	99	35 (15.5)	0	96	92	0 (—)	0	99	96	1 (0.5)	0
Lithuania (1992)	15–16 mos	6–7 yrs	97	95	118 (35.3)	0	94	92	0 (—)	0	93	93	0 (—)	0
Luxembourg (1995)	12 mos	15–23 mos	95	NR	NR	NR	99	86	0 (—)	0	99	90	0 (—)	NR
Malta (1985)	13 mos	3–4 yrs	86	60	6 (14.8)	0	89	91	0 (—)	0	96	95	0 (—)	0
Monaco (1970)	12 mos	16 mos	96	NR	NR	NR	89	79	0 (—)	NR	88	79	0 (—)	0
Montenegro (1994)	13 mos	6 yrs	NR	NR	NR	NR	64	94	0 (—)	NR	42	86	0 (—)	NR
Netherlands (1974)	14 mos	9 yrs	95	93	364 (22.2)	4	95	92	1 (0.6)	0	94	90	0 (—)	0
North Macedonia (1982)	12 mos	6 yrs	96	95	31 (15.0)	NR	89	93	1 (0.5)	NR	75	94	0 (—)	NR
Norway (1978)	15 mos	11 yrs	90	91	1 (0.2)	0	95	91	0 (—)	0	97	95	0 (—)	0
Poland (1994)	13–15 mos	10 yrs	98	90	7,946 (207.1)	0	96	94	2,029 (52.5)	–NR	93	92	292 (7.7)	NR
Portugal (1984)	12 mos	5 yrs	93	87	227 (21.6)	0	98	95	8 (0.7)	4	99	96	0 (—)	0
Moldova (2002)	12 mos	7 yrs	97	98	32 (7.7)	0	89	90	0 (—)	0	97	95	0 (—)	0
Romania (2004)	12 mos	5 yrs	97	96	6,801 (317.5)	1	86	80	18 (0.9)	4	90	76	4 (0.2)	7
Russia (2000)	12 mos	6 yrs	99	97	144,985 (1,009.0)	2	98	97	14 (0.1)	1	98	97	34 (0.2)	0
San Marino (1995)	15 mos	10 yrs	94	94	1 (430.3)	0	84	86	0 (—)	NR	86	79	0 (—)	0
Serbia (1993)	24 mos	7 yrs	96	98	153 (16.6)	NR	86	86	0 (—)	NR	87	91	0 (—)	0
Slovakia (1985)	14 mos	10 yrs	98	98	1 (0.2)	0	95	98	0 (—)	0	96	98	0 (—)	0
Slovenia (1972)	12 mos	5 yrs	94	99	0 (—)	0	94	96	0 (—)	0	94	94	0 (—)	0
Spain (1978)	12 mos	3–4 yrs	97	92	592 (13.4)	5	96	94	4 (0.1)	NR	98	94	4 (0.1)	0
Sweden (1982)	18 mos	6–8 yrs	96	95	0 (—)	0	98	95	0 (—)	0	97	95	0 (—)	0
Switzerland (1973)	12 mos	15–23 mos	87	71	NR	0	94	87	3 (0.4)	0	95	90	0 (—)	NR
Tajikistan (2009)	12 mos	6 yrs	85	92	1,231 (181.3)	NR	97	94	1 (0.1)	NR	98	97	0 (—)	NR
Turkey (1998)	12 mos	6 yrs	91	98	2,245 (33.1)	2	97	86	0 (—)	0	97	88	45 (0.5)	0
Turkmenistan (2007)	12–15 mos	6 yrs	98	99	498 (104.7)	NR	99	99	0 (—)	0	99	99	0 (—)	0
Ukraine (2001)	12 mos	6 yrs	96	96	22,248 (4,743.9)	0	56	57	0 (—)	NR	93	92	138 (3.1)	0
United Kingdom (1970)	12 mos	3–4 yrs	82	75	35 (0.6)	1	93	89	10 (0.2)	2	91	87	3 (<0.1)	0
Uzbekistan (2006)	12 mos	6 yrs	99	81	440 (16.6)	0	99	99	0 (—)	0	98	99	0 (—)	0
**Total**	**—**	**—**	**—**	**—**	**206,359 (234.9)**	**16**	**89**	**94**	**2,457 (2.7)**	**19**	**91**	**96**	**620 (0.7)**	**8**

**Abbreviations:** ASUs = annual status update reports; CRS = congenital rubella syndrome; NR = no report; RCV = rubella-containing vaccine; RCV1 = first RCV dose; RCV2 = second RCV dose; WHO = World Health Organization.

* Based on data from WHO–UNICEF Estimates of National Immunization Coverage, WHO–UNICEF Joint Reporting Form and ASUs from the Regional Verification Commission for measles and rubella elimination process. https://apps.who.int/immunization_monitoring/globalsummary/timeseries/tswucoveragedtp3.html

^†^ Includes clinically compatible cases, laboratory confirmed cases, and epidemiologic linkage cases, as reported to the WHO European Regional Office database. https://www.euro.who.int/en/health–topics/disease–prevention/vaccines–and–immunization/publications/surveillance–and–data/who–epidata

^§^ Cases per 1 million population.

^¶^ WHO vaccine-preventable diseases monitoring systems. Last updated July 15, 2020. https://apps.who.int/immunization_monitoring/globalsummary/schedules?sc%5Br%5D%5B%5D=EURO&sc%5Bd%5D=&sc%5Bv%5D%5B%5D=MMR&sc%5Bv%5D%5B%5D=MMRV&sc%5Bv%5D%5B%5D=MR&sc%5Bv%5D%5B%5D=RUBELLA&sc%5BOK%5D=OK

## Surveillance Activities

Rubella surveillance data are reported monthly to WHO from all EUR countries either directly or via the European Centre for Disease Prevention and Control. As of 2019, 47 (89%) countries reported case-based rubella surveillance data, and six (11%) countries reported aggregate data. Suspected rubella cases are investigated and classified as laboratory-confirmed, epidemiologically linked to a laboratory-confirmed case, clinically compatible, or discarded^§^ (*6*). The WHO European Measles and Rubella Laboratory Network provides laboratory confirmation and genotyping of rubella virus isolates from patients with reported cases (*7*). Important rubella case-based surveillance performance indicators include 1) the number of suspected cases discarded as nonmeasles or nonrubella (target: ≥2 per 100,000 population); 2) the percentage of case investigations conducted within 48 hours of report (target: ≥80%); 3) the percentage of suspected cases (excluding those that are epidemiologically linked) with an adequate specimen collected within 28 days of rash onset and tested in a WHO-accredited or proficient laboratory (target: ≥80%); and 4) the percentage of cases for which the origin of infection (i.e., the source of the virus) is determined (target: ≥80%) (*6*). During 2014–2019, the number of EUR countries that met the target for suspected cases discarded as nonrubella at the national level, a measure of surveillance sensitivity, increased from six (11%) in 2014 to 13 (25%) in 2019. From 2014 to 2019, the number of countries achieving the targets for timely investigations of suspected cases and adequate specimen collection increased from 29 (54%) to 34 (64%) and from 11 (21%) to 35 (65%), respectively.

## Rubella Incidence and Genotypes

**FIGURE 1:**
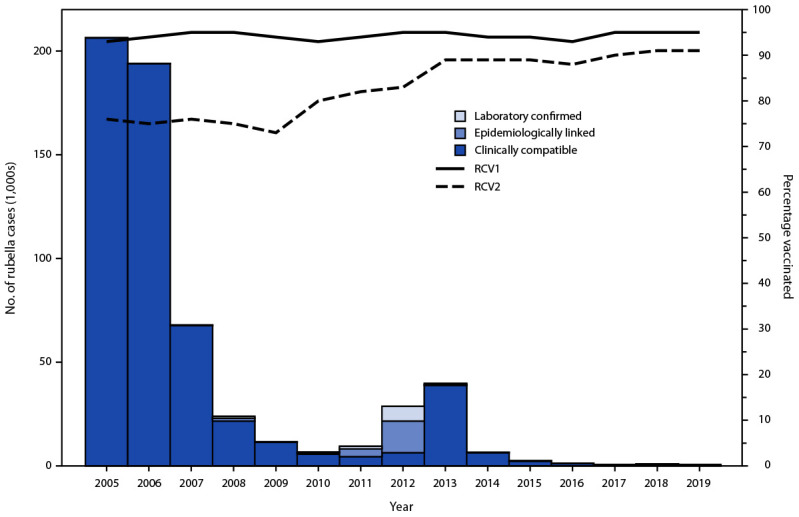
Confirmed rubella cases,* by year of rash onset and confirmation method, and estimated regional coverage through routine vaccination programs with first and second doses of rubella-containing vaccine^†^ — World Health Organization European Region, 2005–2019 **Abbreviations:** RCV1 = first dose of a rubella-containing vaccine; RCV2 = second dose of a rubella-containing vaccine; WHO = World Health Organization. * Confirmed rubella cases reported by countries and areas to WHO. A case of rubella was laboratory-confirmed when rubella-specific immunoglobulin M antibody was detected in serum, rubella-specific RNA was detected by polymerase chain reaction testing, or rubella virus was isolated in cell culture in a person who had not been vaccinated in the 30 days before rash onset; a case of rubella was confirmed by epidemiologic linkage when a case of febrile rash illness was linked in time and place to a laboratory-confirmed rubella case. clinically compatible rubella case is a suspected case that has not been adequately tested by laboratory and has not been epidemiologically linked to a confirmed rubella case. ^†^ WHO and United Nations Children’s Fund Estimates of National Immunization Coverage, July 15, 2020. https://apps.who.int/immunization_monitoring/globalsummary/timeseries/tswucoveragedtp3.html

During 2005–2019, annual regional rubella incidence decreased from 234.9 per 1 million population (206,359 cases) in 2005 to 0.67 per 1 million population (620 cases) in 2019 (Table) (Figure 1). The highest rubella incidences in 2019 were in Poland, with 7.7 cases per 1 million population (292 cases) and Ukraine, with 3.1 cases per 1 million population (138 cases). The last documented rubella outbreaks in the region during this period were reported from Romania in 2012 (1,873 cases) and Poland in 2013 (38,548 cases). During 2013–2019, reported rubella cases dropped substantially and occurred as sporadic cases or small clusters, rather than as ongoing transmission or outbreaks. In 2005, 16 CRS cases were reported from seven countries; in 2019, only eight CRS cases were reported from Azerbaijan (one case) and Romania (seven cases) (Table). Genotyping of rubella viruses in the region has been limited; during 2005-2019, EUR reported only 143 rubella virus sequences to the WHO global rubella nucleotide sequence database, providing insufficient data to contribute substantially to determination of elimination of endemic rubella virus transmission nationally or regionally.

## Regional Verification Commission and Progress Toward Elimination

**FIGURE 2:**
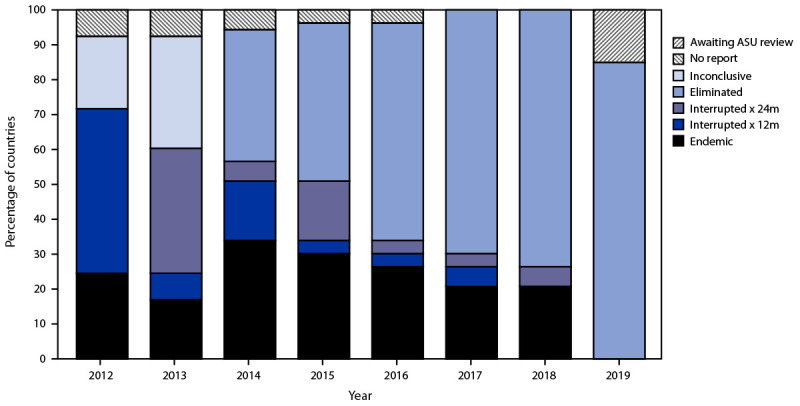
Rubella elimination status, by verification category* — World Health Organization European Region, 2012–2019 **Abbreviations:** ASU = annual status update; WHO = World Health Organization * Verification categories as determined by the European Verification Commission for Measles and Rubella (EU-RVC). *Elimination:* Sustained interruption of rubella virus chains of transmission for >36 months*. Interrupted x 24m:* Sustained interruption of rubella virus chains of transmission for >24 months. *Interrupted x 12m:* Sustained interruption of rubella virus chains of transmission for >12 months. *Endemic:* Continuous transmission of rubella virus(es) that persist for >12 months. *Inconclusive:* ASU submitted but the EU-RVC was unable to reach a conclusion regarding elimination. *No report:* No ASU submitted for review by the EU-RVC. *Awaiting ASU review:* ASU not submitted to the EU-RVC but with plans to submit. Delays in submission of 2019 ASU were because of COVID-19 response activities and limited staff member availability; additional time and support has been provided to these countries by the WHO Regional Office.

The European Regional Verification Commission for Measles and Rubella Elimination (EU-RVC) was established in 2011 to evaluate the status of measles and rubella elimination in EUR countries based on documentation submitted annually by national verification committees (*8**,**9*). By the end of 2019, 45 (85%) countries had sustained interruption for ≥36 months and were verified to have eliminated endemic rubella virus transmission (Figure 2).

## Discussion

After the 2005 WHO Regional Committee resolution that called for regional elimination of rubella and CRS, EUR has made substantial progress with high reported regional RCV coverage, low rubella incidence, and verification that nearly all countries have achieved elimination of endemic transmission of rubella viruses. EUR countries have shown their commitment to the elimination of measles and rubella and CRS with the Regional Committee resolution in 2005 and reaffirmed that commitment with resolutions in 2010 and 2014 (*1*–*3*). All EUR countries have longstanding 2-dose routine RCV immunization schedules that have provided a substantial foundation for establishing population immunity against rubella viruses. Regional rubella coverage with the first and second doses of RCVs has exceeded 93% and 80%, respectively, for the past 10 years. Immunization gaps account for sporadic rubella cases reported in 2019 and have not resulted in confirmed ongoing transmission of rubella viruses.

One of the considerable challenges with verifying rubella elimination has been classifying the large number of clinically compatible rubella cases. In part because rubella is typically a mild disease, a laboratory test is not always performed to confirm a diagnosis, and testing varies considerably across the region according to public health systems, clinical practice, and laboratory capacities. Without a confirmatory laboratory test result, suspected cases are classified as clinically compatible. Increased testing and efficiencies of subnational laboratory networks have contributed to improvements in the surveillance indicators and case classification, but additional efforts are needed.

Rubella population immunity estimates across the region based on longstanding national immunization coverage rates are well above the rubella herd immunity threshold of 83%–86%, which should disrupt chains of transmission and stop or slow the spread of disease (*10*). However, subnational immunization gaps pose a risk for importation and circulation of rubella viruses. Efforts to eliminate endemic transmission of rubella have benefited substantially from the national and subnational outbreak response immunization campaigns for measles cases with combined measles- and rubella-containing vaccines, particularly for large measles outbreaks during 2017–2020.

Retrospective rubella reviews for the verification process provided an opportunity to focus on the specific documentation needed to support the elimination of endemic rubella virus transmission and consisted of an in-depth analysis of information provided in the annual status update reports and supplementary information from national immunization programs, surveillance networks, and the measles-rubella laboratory networks. The retrospective reviews were shared with the EU-RVC members on an ad hoc basis and provided an opportunity to ask for clarification or additional information before drawing conclusions about a country’s rubella elimination status. Data to complete the rubella retrospective reviews for the last four countries with endemic rubella transmission (Bosnia and Herzegovina, Italy, Poland, and Ukraine) are being collected, analyzed, and formatted for submission to the RVC for their determination.

The findings in this report are subject to at least three limitations. First, sensitivity of integrated measles and rubella surveillance might be lower for rubella than it is for measles because it is a milder illness, resulting in possible detection of fewer cases. Second, direct comparisons between countries might not be valid because of variations in their historic approach to diagnosis, case investigation, and laboratory testing. Finally, the region comprises countries with widely different population sizes and compositions, making regional successes and challenges difficult to measure.

Substantial progress toward rubella elimination has been made in EUR. Verification of elimination is nearly complete, which would make EUR the second WHO region to achieve rubella elimination, the first being the Region of the Americas. Sustaining regional rubella elimination will require maintaining high coverage with RCVs through routine immunization programs at the national and subnational levels, offering supplementary rubella vaccination to susceptible adults, maintaining high-quality laboratory-supported rubella and CRS surveillance for outbreak detection and response, and a fully functioning Regional Verification Commission. Because of the COVID-19 pandemic, additional efforts might be needed to strengthen surveillance systems and fill in the immunity gaps.

**Summary**

**What is already known about this topic?**

In 2000, estimated coverage with the first dose of a rubella-containing vaccine (RCV1) in the World Health Organization European Region (EUR) was 60%, and 621,039 rubella cases were reported (incidence = 716.9 per 1 million population).

**What is added by this report?**

During 2005–2019, estimated EUR RCV1 coverage was 93%–95%. In 2019, 31 (58%) countries had achieved ≥95% RCV1 coverage. Rubella incidence declined from 234.9 cases per 1 million population in 2005 to 0.7 cases per 1 million population by 2019.

**What are the implications for public health practice?**

Sustaining regional rubella elimination will require maintaining high coverage with rubella-containing vaccines through routine immunization, offering supplementary rubella vaccination to susceptible adults, and maintaining high-quality surveillance.

## Footnotes

* The European Region, with a population of approximately 900 million, is one of six WHO regions and comprises 53 countries: Albania, Andorra, Armenia, Austria, Azerbaijan, Belarus, Belgium, Bosnia and Herzegovina, Bulgaria, Croatia, Cyprus, Czechia, Denmark, Estonia, Finland, France, Georgia, Germany, Greece, Hungary, Iceland, Ireland, Israel, Italy, Kazakhstan, Kyrgyzstan, Latvia, Lithuania, Luxembourg, Malta, Moldova, Monaco, Montenegro, Netherlands, North Macedonia, Norway, Poland, Portugal, Romania, Russia, San Marino, Serbia, Slovakia, Slovenia, Spain, Sweden, Switzerland, Tajikistan, Turkey, Turkmenistan, Ukraine, United Kingdom, and Uzbekistan.

† In November 2020, Tajikistan finalized transition from measles-rubella vaccine to MMR, resulting in the entire region using MMR.

§ A suspected rubella case is one with signs and symptoms consistent with rubella clinical criteria: 1) maculopapular rash, and 2) cervical, suboccipital or post-auricular adenopathy, or arthralgia/arthritis. A laboratory-confirmed rubella case is a suspected case that meets the laboratory criteria for rubella surveillance case confirmation. An epidemiologically linked rubella case is a suspected case that has not been adequately tested by laboratory and that was in contact with a laboratory-confirmed rubella case 12–23 days before onset of the disease. A clinically compatible rubella case is a suspected case that has not been adequately tested by laboratory and has not been epidemiologically linked to a confirmed rubella case. A discarded case is a suspected case that was investigated and discarded, either through negative results of adequate laboratory testing for rubella or by an epidemiological link to a laboratory-confirmed case to another disease; in addition, IgM-positive cases in recent vaccine recipients can be discarded if they meet all of the following criteria: 1) history of vaccination with relevant vaccine 7 days to 6 weeks before specimen collection, 2) onset of rash 7–14 days after vaccination, 3) no evidence of virus transmission revealed by active search in community, and 4) no history of travel to areas in which the virus is known to be circulating.
