# *Erratum:* Vol. 70, No. 23

**DOI:** 10.15585/mmwr.mm7025a4

**Published:** 2021-06-25

In the report “Progress Toward Rubella Elimination — World Health Organization European Region, 2005–2019,” multiple errors occurred. On page 833, the second author’s name should have read, “**Dragan Jankovic**.” On page 833, in the fourth line of the second column, the sentence should have read, “During 2005–2019, estimated regional coverage with RCV1 was 93%–95%, and in 2019, **30 (57%)** countries achieved ≥95% coverage with the RCV1.” On page 834, in the 11th line of first paragraph under “Immunization Activities,” the sentence should have read, “During 2005–2019, estimated regional coverage with RCV1 was 93%–95%, and in 2019, **30 (57%)** countries achieved ≥95% coverage with the first dose of RCV.” On page 835, in Table 1, in the “Total” line, for the % Coverage columns for 2005, the percentages for RCV1 and RCV2 should have read, “**93**” and “**76**,” respectively; for the % Coverage columns for 2015, the percentages for RCV1 and RCV2 should have read, “**94**” and “**89**,” respectively; and for the % Coverage columns for 2019, the percentages for RCV1 and RCV2 should have read, “**95**” and “**91**,” respectively. On page 838, the second sentence in the “What is added by this report?” paragraph of the Summary box should have read, “In 2019, **30 (57%)** countries had achieved ≥95% RCV1 coverage.”

